# Comparison Between Movement-Based and Task-Based Mirror Therapies on Improving Upper Limb Functions in Patients With Stroke: A Pilot Randomized Controlled Trial

**DOI:** 10.3389/fneur.2019.00288

**Published:** 2019-03-26

**Authors:** Zhongfei Bai, Jiaqi Zhang, Ziwei Zhang, Tian Shu, Wenxin Niu

**Affiliations:** ^1^Department of Occupational Therapy, Shanghai YangZhi Rehabilitation Hospital (Shanghai Sunshine Rehabilitation Center), Tongji University School of Medicine, Shanghai, China; ^2^Department of Rehabilitation Sciences, Tongji University School of Medicine, Shanghai, China; ^3^Department of Rehabilitation Sciences, The Hong Kong Polytechnic University, Kowloon, Hong Kong

**Keywords:** mirror therapy, task-oriented training, upper limb, stroke, rehabilitation

## Abstract

**Objective:** The aim of this trial was to compare the effect of movement-based mirror therapy (MMT) and task-based mirror therapy (TMT) on improving upper limb functions in patients with stroke.

**Methods:** A total of 34 patients with sub-acute stroke with mildly to moderately impaired upper limb motor functions. The participants were randomly allocated to one of three groups: MMT, TMT, and conventional treatment (CT). The MMT group underwent movement-based mirror therapy for around 30 min/day, 5 days/week, for 4 weeks, whereas the TMT group underwent dose-matched TMT. The CT group underwent only conventional rehabilitation. The MMT and TMT groups underwent CT in addition to their mirror therapy. Blinded assessments were administered at baseline and immediately after the intervention. Upper limb motor functions, measured using Fugl-Meyer Assessment-upper extremity (FMA-UE), Wolf Motor Function Test (WMFT), and hand grip strength; upper limb spasticity, measured using the modified Ashworth scale (MAS); and activities of daily living, measured using the modified Barthel index (MBI).

**Results:** A significant time-by-group interaction effect was noted in FMA-UE. *Post-hoc* analysis of change scores showed that MMT yielded a better effect on improving FMA-UE than the other two therapies, at a marginally significant level (*P* = 0.050 and 0.022, respectively). No significant interaction effect was noted in WMFT, hand grip strength, MAS, and MBI.

**Conclusion:** Both MMT and TMT are effective in improving the upper limb function of patients with mild to moderate hemiplegia due to stroke. Nevertheless, MMT seems to be superior to TMT in improving hemiplegic upper extremity impairment. Further studies with larger stroke cohorts are expected to be inspired by this pilot trial.

**Trial registration number:** No. ChiCTR1800019043 (http://www.chictr.org.cn/index.aspx)

## Introduction

Mirror therapy (MT) has been shown to be a useful intervention for rehabilitation of upper limb functions following stroke, since the first attempt by Altschuler et al. (1). The neural correlate of MT remains under investigation. Three main theories explaining the neural mechanism underlying the clinical efficacy of MT have been proposed (2).

The first theory hypothesizes that the neural correlate of MT is the mirror neuron system (MNS), which is defined as a class of neurons that fire during action observation and action execution (3). It is assumed that the MNS can be triggered when people are observing mirror visual feedback (MVF) generated in MT (4, 5). The affected cortical motor system can be accessed via the MNS owing to their functional connections (6). The second theory, supported by several studies with transcranial magnetic stimulation (TMS), suggests that a potential neural mechanism underlying the effect of MT can be the recruitment of the ipsilesional corticospinal pathway. Indeed, many TMS studies have demonstrated the increment of motor-evoked potentials of the ipsilesional primary motor cortex in participants with stroke when viewing MVF (7), which indicates a facilitatory effect of MVF on the ipsilesional corticospinal pathway. The last theory attributes the effect of MT to the compensation of restricted proprioception input from the affected limb and the enhancement of attention toward the paretic upper limb (8), which may contribute to the reduction of the learned non-use in patients with stroke (1).

A substantial number of randomized controlled trials (RCTs) have demonstrated that MT is useful in improving upper limb functions after stroke (9–12). A recently published meta-analytic review identified a moderate level of evidence supporting the effects of MT on improving upper limb motor functions (Hedges' *g* = 0.47) and activities of daily living (ADLs) (Hedges' *g* = 0.48) in patients with stroke (13). In the meta-analysis (13), the heterogeneity of conducting MT was obvious across studies. One major category of MT is movement-based MT (MMT), in which participants practice simple movements such as wrist flexion and extension, or finger flexion and extension, with their unaffected hands when viewing the MVF generated by a physical mirror placed at their mid-sagittal plane (14–16). Another category of MT is task-based MT (TMT), in which participants perform specific motor tasks with their unaffected hands, such as squeezing sponges, placing pegs in holes, and flipping a card, while they are viewing the MVF (12, 17). In some studies, researchers applied MMT in the first few sessions and subsequently applied TMT in the following sessions, constituting a hybrid MT protocol (9, 10, 18). MMT and TMT were also described as intransitive and transitive movements in some studies (9, 10). However, a sub-group meta-analysis comparing MMT and TMT was not carried out in the meta-analysis study (13).

Initially, MMT was used for alleviating phantom pain after amputation and for treating upper limb hemiplegia after stroke (1, 19). Subsequently, the effect of MMT in stroke upper limb rehabilitation has been systematically investigated by many clinical trials (14–16, 20). Arya et al. were the first to compare the effects of TMT with those of conventional rehabilitation on upper limb motor recovery after stroke, and they found a superior effect of TMT (12). The main rationale that Arya et al. mentioned was that the response of the MNS was better for object-directed actions than for non-object actions (12, 21). In a recent study comparing the effects of action observation training and MT on gait and balance in patients with stroke, the results showed that action observation training had significantly better effects on the improvement of balance functions than MT (22), indicating that action observation may be different from MT in terms of their neural mechanisms. In other studies in which TMT was introduced or combined with MMT, the authors did not explain why they employed TMT (9–11).

Thus far, no RCT has systematically investigated the difference between the effects of MMT and TMT. Therefore, we aimed to conduct an RCT to directly compare the effect of MMT and TMT, on improving hemiplegic upper limb motor functions, spasticity, and ADLs, in a group of patients with stroke.

## Methods

### Study Design and Procedure

This study was designed as an assessor-blinded RCT. A computer random-number generator was used to generate the random sequence for group allocation. All participants were informed about the purpose and procedures of this study and provided signed informed written consent before their participation. An investigator who was blinded to patient selection kept the random sequence and allocated the participants to one of three groups: MMT, TMT, or conventional treatment (CT), at a 1:1:1 ratio. Three assessors and four occupational therapists who performed the outcome measurements and intervention, respectively, were trained by a senior occupational therapist in stroke rehabilitation before the study. The same assessor (one of three) carried out the pre- and post-test assessments for the same participant. All assessors were blinded to the group allocation. Blinding of the occupational therapists providing interventions and of the participants were impossible because of the nature of MT.

The ethical application of this study was approved by the Research Committee of Shanghai YangZhi Rehabilitation Hospital (Shanghai Sunshine Rehabilitation Center, reference no. YZ2016-021). This study was retrospectively registered in the Chinese Clinical Trial Registry (no. ChiCTR1800019043).

### Participant Recruitment

All participants were recruited from a rehabilitation hospital between June 7, 2016, and April 11, 2018. The participants were referred for this study by their occupational therapists in charge. A total of 95 post-stroke patients were screened.

Participants who met all of the following criteria were included: (1) a first-ever unilateral ischaemic or haemorrhagic cerebrovascular accident with onset between 1 and 6 months; (2) mild to moderate motor impairment, level 3 to 5 in the Functional Test for the Hemiplegic Upper Extremity (23); (3) mild to moderate degree of spasticity in all joints of the affected upper limb; and (4) sufficient cognitive ability to follow instructions (Mini-mental State Examination score >24).

Patients who met any of the following criteria were excluded: (1) participation in another drug or experimental project within 3 months; (2) aphasia; (3) serious unilateral neglect (Star Cancellation Test ≤ 44/54) or visual field deficiency; (4) any other comorbid neurological diseases except for stroke; and (5) diagnosis of any other neuromuscular or orthopedic disease in the upper extremities.

### Interventions for the CT Group

All patients in the CT group underwent multi-disciplinary rehabilitation training, including customary physiotherapy and occupational therapy. The physiotherapy intervention, lasting for 1–2 h/day, focused on the patients' lower limb motor function and ambulation. Usually, physiotherapists applied muscle stretching before active motor training. Moreover, intensive training for ambulation, consisting of dynamic walking balance and gait patterns, was provided for the included participants. For occupational therapy intervention, the participants underwent 1.5 h of training, including customary upper limb functional training and ADL training. In customary upper limb functional training, the primary principle was to apply individual task-oriented training for the affected arms to enhance muscle strength, endurance, coordination, and functional use.

All participants underwent the interventions 5 days/week, for a total of 4 weeks between the pre-test and post-test. After the post-intervention assessment, they underwent CT as usual until discharge.

### Interventions for the MMT Group

Participants in the MMT group underwent the same physiotherapy as that in the CT group. During the MMT training, the participants were instructed to sit on a chair in front of a table. A mirror on the table was positioned perpendicular to the participants. All jewelleries were removed from the unaffected arms, and the affected hand was positioned behind the mirror, whereas the unaffected hand was placed in the front of the reflective surface. Thereby, the participants were asked to view the reflected upper limbs in the mirror instead of their real upper limb.

Once the training started, the participants were asked to perform some simple movements with the affected upper limb, such as (1) finger tapping, (2) griping and releasing, (3) wrist ulnar and radial derivations, (4) wrist extension and flexion, (5) forearm pronation and supination, (6) elbow extension and flexion, (7) moving the affected arm from the middle position to the lateral side, and (8) lifting the hand up and returning it to the table.

During the practices with the unaffected arm, the participants were instructed to move their affected arm synchronically while viewing the mirror. Each movement was repetitively performed for 3–4 min, with a total of 30 min for 1 MMT session. A 30-s break was allowed intermittently when changing the movements. The task-oriented training for upper limb motor function and ADL training was conducted in the remaining 1 h, which was the same as that in the CT group.

The treatment dose of the MMT group was similar to that of the CT group: 5 days/week, for a total of 4 weeks between the pre-test and post-test. After the post-test, the participants underwent the same training as the CT group until discharge.

### Interventions for the TMT Group

All procedures and setup for the TMT training were the same as those for the MMT training. However, the upper limb movements performed in TMT were tasks instead of simple movements. Six tasks were performed with the affected hand during TMT, including (1) transferring small cubes from the middle position to the lateral side, (2) placing pegs in holes and taking them out, (3) turning over paper cards, (4) placing steel needles in holes, (5) stacking blocks, and (6) putting cups on a shelf. During performing the tasks with the unaffected hand, the participants were instructed to move their affected arm synchronically in the same way while viewing the mirror. Each task should be performed for at least 4 min, with a total of 30 min for one TMT session. The participants could have a 30-s break intermittently when changing tasks. The training in the remaining 1 h and the dose were the same as those in the CT group, 5 days per week, for a total of 4 weeks between the pre-test and post-test. After the post-test, the participants underwent the same training as the CT group until discharge.

### Outcome Measures

Pre-tests were conducted 1 day before the initiation of interventions, and post-tests were conducted 1 day after the completion of all intervention sessions. The primary outcomes were upper limb motor impairment, measured using the Fugl-Meyer Assessment-upper extremity (FMA-UE), and upper limb motor functional performance, measured using the Wolf Motor Function Test (WMFT). The FMA-UE includes 33 items assessing movement, coordination, and reflex actions of the shoulder, elbow, forearm, wrist, and hand joints of the paretic arm. Each item consists of a 3-point scale (0, 1, and 2), with a total maximum score of 66. The FMA-UE has excellent inter-rater reliability (intra-class correlation coefficient [ICC] > 0.95) and test-retest reliability (ICC > 0.95) (24). The minimal clinically important difference (MCID) of FMA-UE is 5.25 (25). The WMFT is a tool for evaluating the functional performance of the hemiplegic upper limb in given tasks. The assessors rated the quality of performance by using a 6-point functional ability scale, and the total score was obtained by summing the individual scores of each item. The total score of the WMFT has excellent inter-rater reliability (ICC = 0.88) and test-retest reliability (ICC = 0.95) (26). The grip strength of the affected hand was assessed with a calibrated Jamar hydraulic hand dynamometer (model SH5001; Saehan Corp, Masan, Korea).

The secondary outcomes included the muscle tone of the affected arm (i.e., biceps) and hand (i.e., wrist flexor) and ADLs, measured using the modified Ashworth scale (MAS) and modified Barthel index (MBI), respectively. The MAS measures muscle tone during passive soft-tissue stretching, with moderate inter-rater (kappa = 0.51) and intra-rater (kappa = 0.60) reliabilities (27). The MBI consists of 10 common daily activities, including feeding, bathing, grooming, dressing, bowel control, bladder control, toileting, chair transfer, ambulation, and stair climbing, with excellent inter-rater reliability (kappa > 0.80) (28) and concurrent validity (Functional Independence Measure, *r* = 0.92) (29).

### Sample Size Calculation

As no study comparing MMT and TMT has been performed, we cannot set an estimated effect size for our sample size calculation. We reviewed all published clinical trials on MT, and the sample size of most studies ranged from 10 to 20 patients in each group. Therefore, we planned to recruit 15 patients in each group for this pilot RCT considering the exploratory nature of the present preliminary study.

### Statistical Analysis

Data analysis was conducted using SPSS version 23.0 (SPSS Inc., Madison, WI, USA). Demographic characteristics and pre-test scores were compared using analysis of variance (ANOVA) for continuous and ordinal data, or Fisher's exact test for categorical data. Two-way repeated-measures ANOVA, with time effect, group effect, and time-by-group interaction effect, was used to assess within-group and between-group differences. If any significant difference was noted in the pre-test score of any dependent variable, one-way analysis of covariance with the pre-test score as covariate was employed instead. The level of significance was set at *P* < 0.05 (2-tailed). *Post-hoc* analysis was performed when any significant interaction effect was noted in two-way repeated ANOVA, by comparing all pairs of mean change scores between groups by using one-way ANOVA with pairwise comparisons. Bonferroni correction was used in *post-hoc* analysis to avoid the inflation of type I errors. The level of significance was set at *P* < 0.017 (0.05/3; 3 = number of paired comparisons) after Bonferroni correction.

## Results

**Figure 1:**
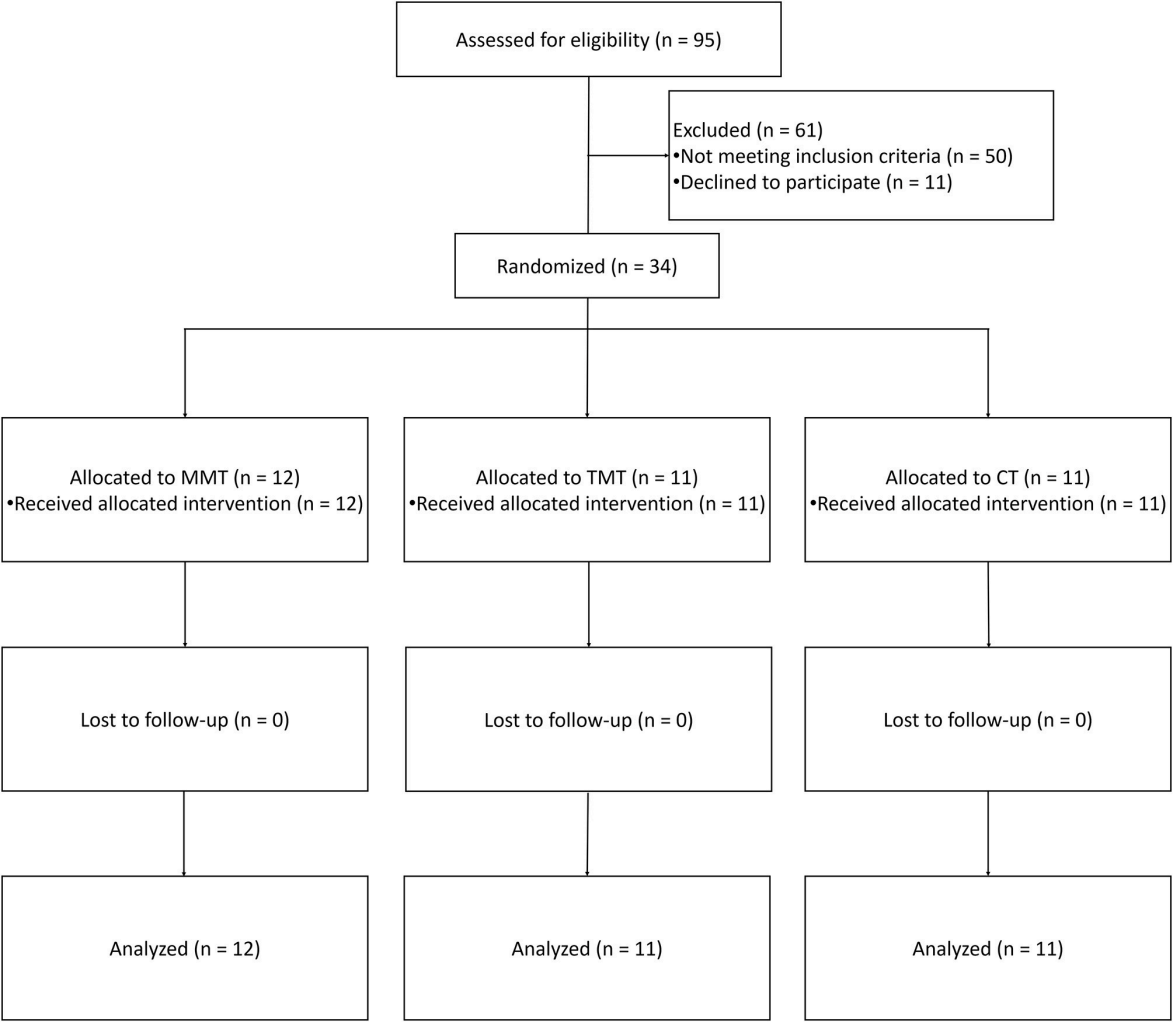
Flowchart of the study. MMT, movement-based mirror therapy; TMT, task-based mirror therapy; CT, conventional treatment.

The patient recruitment process is presented in Figure 1. From a pool of 95 patients with sub-acute stroke, a total of 34 inpatients (MMT = 12, TMT = 11, and CT = 11) were included in the present study. No patient dropped out from this trial. We cannot recruit the expected number of patients owing to the limited time of this trial. The mean age of participants in MMT, TMT, and CT were 56.08 ± 13.61, 54.36 ± 11.56, and 58.27 ± 15.44 years, respectively. Higher percentage of male patients than female patients were enrolled in all groups, 75.00% for MMT, 90.90% for TMT, and 54.55% for CT. For the type of stroke, ischaemic stroke is more common than haemorrhagic stroke, 75.00% for MMT, 63.64% for TMT, 81.81% for CT. The time after stroke onset in CT group is higher than MMT and TMT, but the statistical analysis was not significant (*P* = 0.19). More comparisons of the demographic characteristics and pre-test scores are presented in Table 1. There were no significant differences among the three groups in demographic characteristics and baseline assessments.

**Table 1:** Comparisons of demographic characteristics and baseline assessments.

	**MMT (*n* = 12)**	**TMT (*n* = 11)**	**CT (*n* = 11)**	***F* or *X^**2**^***	***P*-value**
**DEMOGRAPHICS**					
Age (years)	56.08 (13.61)	54.36 (11.56)	58.27 (15.44)	0.23	0.80
Male sex, *n* (%)	9 (75.00%)	10 (90.90%)	6 (54.55%)	3.54	0.17
**STROKE CHARACTERISTICS**					
Time after stroke (days)	61.92 (35.35)	60.00 (44.41)	93.45 (59.75)	1.76	0.19
Side of hemiplegia
Left, *n* (%)	6 (50.00%)	3 (27.27%)	6 (54.55%)	1.91	0.47
Right*, n* (%)	6 (50.00%)	8 (72.72%)	5 (45.45%)		
**TYPE OF STROKE**					
Ischaemic, *n* (%)	9 (75.00%)	7 (63.64%)	9 (81.81%)	0.99	0.72
Haemorrhagic, *n* (%)	3 (25.00%)	4 (36.36%)	2 (18.18%)		
Brunnstrom stage (arm)	3.25 (0.62)	3.55 (1.04)	3.00 (0.45)	1.50	0.24
Brunnstrom stage (hand)	3.42 (1.24)	3.64 (1.75)	4.09 (0.94)	0.74	0.49
FMA-UE	34.25 (12.21)	37.55 (14.19)	35.36 (10.62)	0.21	0.81
WMFT	29.08 (7.38)	34.55 (9.45)	26.09 (9.72)	2.56	0.09
Grip strength	4.30 (3.43)	5.37 (5.93)	4.59 (5.46)	0.14	0.87
MBI	66.25 (17.73)	60.45 (18.36)	62.27 (16.49)	0.33	0.72
MAS (arm)	1.29 (0.26)	1.18 (0.50)	1.05 (0.61)	0.76	0.48
MAS (hand)	1.04 (0.58)	0.86 (0.60)	1.00 (0.59)	0.28	0.76

*Values are represented as n (%) or mean (standard deviation). MMT, movement-based mirror therapy; TMT, task-based mirror therapy; CT, conventional treatment; FMA-UE, Fugl-Meyer Assessment-upper extremity; WMFT, Wolf Motor Function Test; MBI, modified Barthel index; MAS: modified Ashworth scale*.

**Figure 2:**
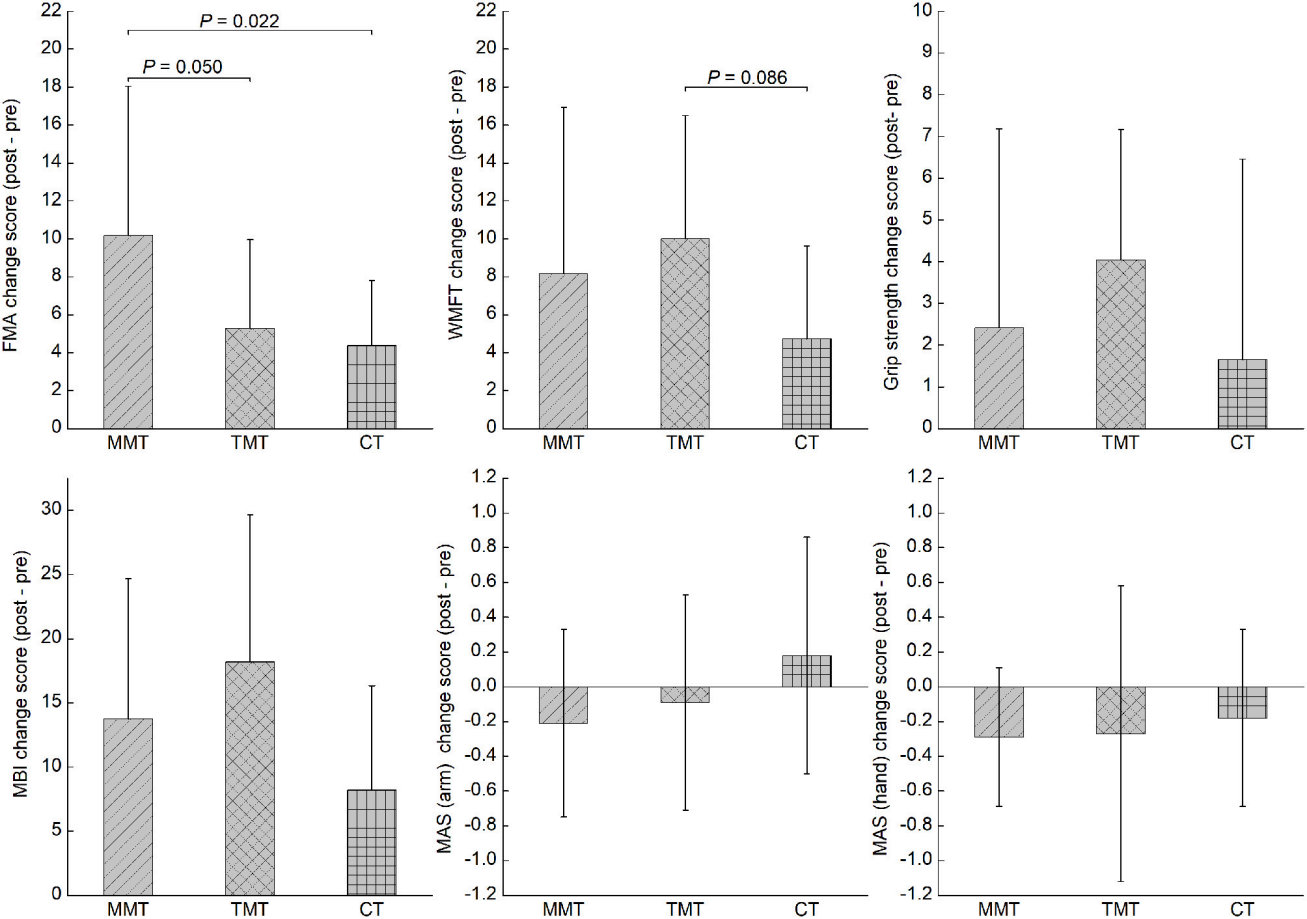
The comparisons in all outcome measures across the three groups. MMT, movement-based mirror therapy; TMT, task-based mirror therapy; CT, conventional treatment; FMA-UE, Fugl-Meyer Assessment-upper extremity; WMFT, wolf motor function test; MBI, modified Barthel index; MAS, modified Ashworth Scale.

Table 2 shows the descriptive data and statistical analysis of the patients' functional outcomes. With respect to our primary outcomes, all patients showed significant improvement in FMA-UE (*F* = 44.85, *P* < 0.001), WMFT (*F* = 40.69, *P* < 0.001), and grip strength (*F* = 13.22, *P* = 0.001), as indicated by the significant time effects. Only 1 significant time-by-group interaction effect was noted in FMA-UE (*F* = 3.44, *P* = 0.045). *Post-hoc* analysis showed that the change score of FMA-UE in the MMT group was significantly higher than that in the TMT and CT groups (*P* = 0.050 and 0.022, respectively). However, they did not survive a Bonferroni correction at a significance level of 0.017 (Figure 2). The number of participants who exceeded the MCID of FMA-UE was 10 (83.3%), 5 (45.5%), and 4 (36.3%) in the MMT, TMT, and CT groups, respectively. Both the MMT and TMT groups tended to yield a higher change score of WMFT than the CT group. However, *post-hoc* analysis showed that the comparisons were not significant (*P* = 0.246 and 0.086, respectively).

**Table 2:** Difference in outcome measurements between groups at pre- and post-test.

	**MMT (*****n*** **=** **12)**			**TMT (*****n*** **=** **11)**			**CT (*****n*** **=** **11)**			**Between-group interaction**	
	**Pre-test**	**Post-test**	**Mean difference**	**Pre-test**	**Post-test**	**Mean difference**	**Pre-test**	**Post-test**	**Mean difference**	***F*-value**	***P*-value**
FMA-UE	34.25 (12.21)	44.42 (12.89)	10.17 (7.88)	37.55 (14.19)	42.82 (13.48)	5.27 (4.69)	35.36 (10.62)	39.73 (11.79)	4.36 (3.44)	3.44	0.045^#^
WMFT	29.08 (7.38)	37.25 (10.91)	8.17 (8.76)	34.55 (9.54)	44.55 (14.17)	10.00 (6.50)	26.09 (9.72)	30.82 (12.71)	4.73 (4.90)	1.63	0.213
Grip strength	4.30 (3.43)	6.71 (5.19)	2.41 (4.77)	5.37 (5.93)	9.41 (7.16)	4.04 (3.13)	4.59 (5.46)	6.25 (9.42)	1.65 (4.81)	0.87	0.428
MBI	66.25 (17.73)	80.00 (13.48)	13.75 (10.90)	60.45 (18.36)	78.64 (12.06)	18.18 (11.46)	62.27 (16.49)	70.45 (19.93)	8.18 (8.15)	2.61	0.090
MAS (arm)	1.29 (0.26)	1.08 (0.56)	−0.21 (0.54)	1.18 (0.51)	1.09 (0.63)	−0.09 (0.62)	1.05 (0.61)	1.23 (0.68)	0.18 (0.68)	1.20	0.316
MAS (hand)	1.00 (0.59)	0.75 (0.45)	−0.29 (0.40)	0.86 (0.60)	0.59 (0.58)	−0.27 (0.85)	1.00 (0.59)	0.81 (0.68)	−0.18 (0.51)	0.104	0.901

*Values are represented as mean (standard deviation). MMT, movement-based mirror therapy; TMT, task-based mirror therapy; CT, conventional treatment; FMA-UE, Fugl-Meyer Assessment-upper extremity; WMFT, wolf motor function Test; MBI, modified Barthel index; MAS, modified Ashworth Scale*.

# *P < 0.05*.

All groups showed significant improvement in MBI, as indicated by a significant time effect (*F* = 57.29, *P* < 0.001); however, there was an insignificant time-by-group interaction effect (*F* = 2.61, *P* = 0.090). A significant time effect was noted in MAS (hand) (*F* = 5.64, *P* = 0.024) but not in MAS (arm) (*F* = 0.14, *P* = 0.714). However, no significant time-by-group interaction effect was noted in MAS (arm).

## Discussion

The current pilot RCT is the first study to systematically investigate the differential effects of MMT and TMT on improving motor impairment and functional performance in patients with sub-acute stroke. Upper limb motor impairments refer to problems in upper limb motor function and structure, such as limited upper limb movement, whereas upper limb functional performance is defined as the ability to perform a task or action with the upper limbs (30). Our results suggested that MMT seemed to be better than TMT in terms of improving hemiplegic upper limb motor impairment, as indicated by the FMA-UE, among patients with sub-acute stroke. A higher proportion of participants who exceeded the MCID of FMA-UE were noted in the MMT group (83.3%) than in the TMT (45.5%) and CT (36.3%) groups. However, upper limb motor functional performance, grip strength, ADL, and upper limb spasticity changed at similar rates across all groups. The effects of TMT were not significantly different from those of CT, which was against our expectation. We found that TMT tended to gain a higher change score of the WMFT than the CT group indicated by a very low *P*-value (*P* = 0.086). The potential reason for not finding significance was our small sample size. Our sample size calculation for a future study indicated that 23 patients in each group are required to gain a significant difference between TMT and CT in the WMFT at a Bonferroni corrected α error probability of 0.017 and a power of 0.8.

Our results of MMT were line with early studies examining the effects of MMT in patients with stroke. Previous researchers did not propose the concepts of TMT and MMT, but we can classify them into MMT or TMT according to the concepts we proposed. In early studies (15, 16), their MMT protocols were shown effective in motor impairment which was in line with our results. Particularly, in the study by Lee et al. (15), the characteristics of participants, sample size and treatment protocols of both experimental and control groups were consistent with ours. Although our results supported the superior effects of MMT in improving motor impairment, these did not support the effects of MMT in improving functional performance. A previous study found that MMT was also more effective than sham MT in improving functional performance in patients with acute stroke, <1 month since stroke onset (16). Because of the heterogenicity of participants and treatment protocol of control group across studies (16), we cannot draw a firm conclusion about the effects of MMT in functional performance.

For the effects of TMT in motor impairment, our result did not support its superior effects compared to CT, which was not consistent with other two studies (12, 17). In these two studies, they recruited patients with stroke more than 6 months since onset, while our study included participants in sub-acute stage. Therefore, we may postulate that the time since stroke is an essential mediator for the effects of TMT in improving motor impairment. In addition, although 4-week interventions were used in most of the previous MT studies (31), Arya et al. (12) implemented the TMT protocol for 8 weeks. Therefore, a longer intervention may reveal the potential benefits of TMT on upper limb motor impairment, in the case that patients become adapted to mirror-based motor practice. Moreover, participants in Arya et al. (12) had more severe hemiplegia (mean of FMA-UE: 19.71) than ours (mean of FMA-UE: 37.55). Therefore, we cannot draw a firm conclusion of TMT in motor impairment due to these heterogeneities. Meanwhile, although we only observed the effect favoring MMT over TMT in terms of improving upper limb motor impairment (i.e., FMA-UE), we cannot underestimate the effect of TMT. We found that TMT, but not MMT, tended to yield a superior effect to that of CT on enhancing upper limb functional performance (i.e., WMFT) in patients with sub-acute stroke. However, further clinical studies are needed to confirm the preliminary findings.

**Figure 3:**
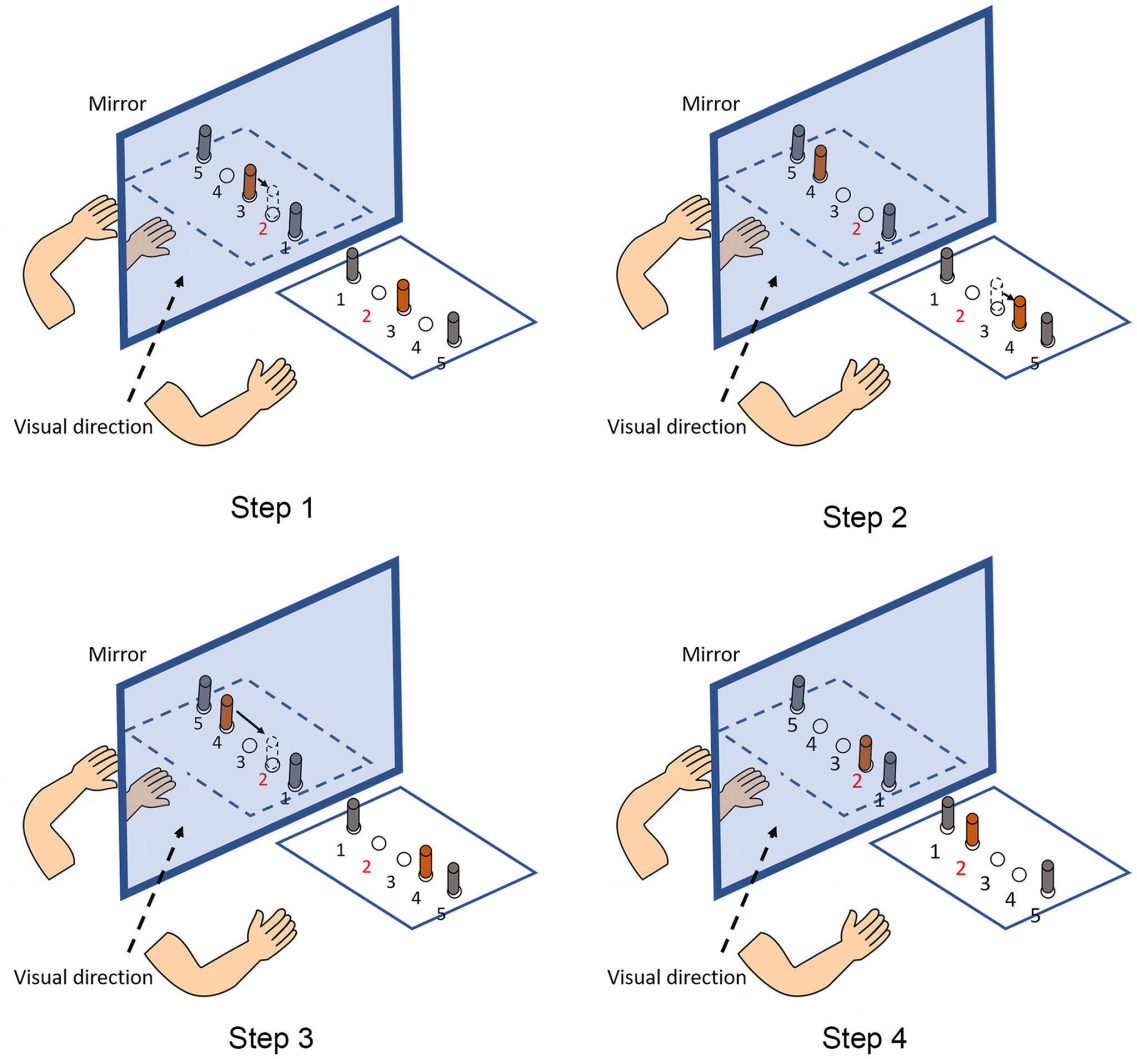
An example of the process of “fault and correction.” The given task is that participants are required to transfer an object placed in the No. 3 hole (in orange color) to the No. 2 hole (Step 1). However, participants usually move the object to the No. 4 hole when they are viewing the mirror reflection (Step 2). Then, participants realize the fault and transfer the object it to the No. 2 hole (Steps 3, 4).

When interpreting the differential effects of MMT and TMT in stroke rehabilitation, the nature of these two MT protocols needs to be considered. The reasons to recommend the application of TMT may include the functional preference of the assumed MNS. Previous evidence has shown that the assumed MNS responds more intensively to object-directed action observation than to non-object action observation (21), because more information from objects for action recognition can be derived by the observer (32, 33). Therefore, it can be hypothesized that TMT may have a superior effect to MMT in triggering the assumed MNS, thereby facilitating the cortical motor system of patients with stroke. In our study, we observed a phenomenon during TMT, which is illustrated in Figure 3. During the training, the participants continuously exhibited the process of “fault and correction” owing to the increased complexity when performing the tasks in MT. The source of the increased complexity was mainly the mirrored spatial location. In the mirrored space, the spatial relationship among objects is opposite relative to real objects. Therefore, the increased complexity requires patients with stroke to manipulate their healthy hands carefully and may be the source of differential effects in motor impairment and functional performance. We must acknowledge that, based on the present clinical study, it remains difficult to explain the differential neural mechanisms underlying MMT and TMT. Further neuroscientific investigations are needed to elucidate the potential differential effect of MMT and TMT on modulating the cortical activity of the motor areas and the potential lateralization change of activation pattern between the left and right hemispheres when receiving different forms of MVF.

## Limitations

Our study is not free of limitations. First, our recruited sample did not reach our expected size. The marginally significant results may have resulted from the limited power. Further studies with larger sample sizes are needed to confirm our preliminary findings. Second, as our participants were limited to hospitalized patients with stroke with mild to moderate hemiplegia, our results may be difficult to generalize to other stroke cohorts, such as community-dwelling stroke survivors. Third, we were not able to conduct follow-up assessment to observe the durability of the training effect, as the length of hospitalization in our medical unit was limited.

## Conclusions

Both MMT and TMT are effective in improving the upper limb function of patients with mild to moderate hemiplegia due to stroke. Nevertheless, MMT seems to be superior to TMT in improving hemiplegic upper extremity motor impairment. Further studies with larger stroke cohorts are expected to be inspired by this pilot trial.

## Data Availability

All datasets generated for this study are included in the manuscript and/or the supplementary files.

## Author Contributions

All authors were involved in drafting the article or revising it critically for important intellectual content, and all authors approved the final version for submission. ZB was responsible for the conception, organization of the research project, drafting the manuscript, and critically reviewing the statistical analysis. JZ was responsible for drafting the manuscript, statistical analysis, and interpretation. ZZ and TS were responsible for the organization and execution. WN was also responsible for study design, statistical analysis, and critically reviewing for the manuscript.

### Conflict of Interest Statement

The authors declare that the research was conducted in the absence of any commercial or financial relationships that could be construed as a potential conflict of interest.
